# Accessing care: qualitative analysis of counseling center therapists’ experiences of transitioning to telehealth

**DOI:** 10.1080/28324765.2025.2470126

**Published:** 2025-03-03

**Authors:** Tatiana Leroy, David Nicholas Top, Russell J. Bailey, Christina Bartholomew, Julia Toomey, Taylor Baker, Josephine Schwalbe, Kirra D. Jensen

**Affiliations:** a Department of Psychology, University of Utah, Salt Lake City, UT, USA; b Student Health Service, Utah Valley University, Orem, UT, USA; c Department of Psychology and Counseling, Utah Valley University, Orem, UT, USA; d School of Social Work, Brigham Young University, Provo, UT, USA; e College of Social Work, University of Utah, Salt Lake City, UT, USA

**Keywords:** Telehealth, COVID-19 pandemic, teletherapy, online therapy services, transition, qualitative research

## Abstract

The study aimed to explore and categorize therapists’ experiences with the transition to telehealth during the COVID-19 pandemic. We conducted in-depth interviews with 13 therapists working at a university counseling center in the United States to more thoroughly understand their experiences. We then employed consensual qualitative research methods within a research team to analyze these data across multiple phases. The findings revealed several key domains, including obstacles and changes in therapy style and relationships, therapists’ attitudes towards telehealth, perceived fit of clients for telehealth, ethical concerns for the practice of telehealth, the need for training and established telehealth protocols, and logistical challenges for implementing telehealth. The results emphasized the need for additional research, training, and development of best practices in telehealth to support therapists in delivering effective and ethical therapy services online.

## Background

Since the advent of the COVID-19 pandemic, much has changed in how people live their lives. Extensive studies have shown that this pandemic has had a detrimental impact on the population’s mental and physical health, including increased levels of psychological distress, irritability, anxiety, depression, and post-traumatic stress symptoms. These outcomes can be attributed to various factors, including longer quarantine duration (Brooks et al., 2020), isolation and loneliness (Armitage & Nellums, 2020), death and grief (Bertuccio & Runion, 2020), and an overall decrease in psychological and physiological well-being due to limited mobility and physical activity (Wang & Boros, 2021).

As the pandemic exacerbated mental and physical health challenges, therapists had to rapidly adapt by transitioning to telehealth (TH), relying on technology to deliver mental health services to clients. According to one study (*n* = 903), only 20% of therapists used a TH format before the COVID-19 outbreak (Reilly et al., 2020). Pre-pandemic research has documented that both practicing and future clinicians often felt hesitant about TH, citing concerns about its efficacy, insufficient training and information, and the perceived challenges in building a therapeutic bond, managing clients in crisis, and ensuring privacy and confidentiality in an online setting (Alleman, 2002; Fenichel et al., 2002; Glueckauf et al., 2018; Lovejoy et al., 2009; Mallen et al., 2005; Perle et al., 2014). It is difficult to imagine this sudden transition going smoothly, considering that prior to the pandemic, neither TH practices nor TH training was comprehensively established within the industry (Perle et al., 2012).

Several post-pandemic studies attempted to qualitatively capture therapists’ experiences during this shift. For example, Full et al. (2024) conducted a qualitative survey with 590 clinicians working in private practice or voluntary sector, which offered a valuable snapshot of therapists’ experiences working online. However, the predominantly UK-based sample (95%) may not accurately represent the experiences of therapists in the US. Additionally, the structured nature of their survey limited opportunities for follow-up questions, which could have elicited deeper insights into some of the ambiguities that emerged in their findings. Similarly, McCoyd et al. (2022) used structured surveys with open-ended text boxes to examine a larger sample of New Jersey-based social workers. Their convenience sample (*n* = 448) was notably older, with two-thirds of the participants in the 40–69 age range and predominantly female (89%). Additionally, their analysis was based on the comments spontaneously offered by participants, lacking the consistency of a more systematic, targeted inquiry.

Several other interview-based qualitative studies examined the experiences of clinicians transitioning to TH (Aafjes van Doorn et al., 2024; Békés et al., 2023; Kotera et al., 2021; Smith & Gillon, 2021). These studies involved relatively smaller numbers of participants (*n* = 5 to *n* = 31) and identified themes regarding therapeutic relationships, therapists’ attitudes, experiences, and challenges faced during this transition. Kotera et al. (2021) primarily focused on highly experienced therapists working within private practices and their attitudes toward TH rather than their experiences. Smith and Gillon (2021) also offered limited information about their participants’ experiences, and their sample was limited to the UK within a specific healthcare system, showing the need for qualitative research in other settings. Aafjes van Doorn et al. (2024) and Békés et al. (2023) focused on similarly small samples with predominantly psychodynamically oriented therapists in a European context.

Overall, we observed the need for qualitative analysis in a US setting that emphasizes short-term treatment models and adapting to the pressures of short-term care through the use of various theoretical orientations (e.g., cognitive behavioral therapy, acceptance and committed therapy, solution-focused, emotion-focused therapy, eclectic, etc.). Similar to managed care settings in the US, there are specific pressures on clinicians to provide care that is brief. Many orientations may be used by clinicians though brevity is often expected in such settings. Furthermore, we identified critical gaps in the existing literature regarding the experiences of therapists working in university counseling settings pertaining to accessibility, the dynamics of short-term care, and the perceived effectiveness of TH in a young adult population.

The existing body of quantitative research with a focus on therapists’ experiences and opinions regarding their transition to TH reveals significant limitations related to the sample characteristics. Most of the recent studies present a demographic skew with over 70% of the samples consisting of female participants (Aafjes van Doorn et al., 2021, 2022; AlRasheed et al., 2022; Barker & Barker, 2022; Békés & Aafjes van Doorn, 2020; Békés et al., 2021; Lin et al., 2021, 2022; Reilly et al., 2020; Sampaio et al., 2021; Sugarman et al., 2021). The findings from these studies may present biases in understanding TH experiences, failing to capture the potential differences between female and male therapists. Additionally, many of these studies, including those by Lin et al. (2021) and Aafjes-van Doorn et al. (2021, 2022), involved predominantly mid-to-late career therapists who may experience the challenges of transitioning to TH differently than trainees and newly licensed therapists. Other studies drew participants from specific settings, such as private practice (Aafjes van Doorn et al., 2021; Lin et al., 2022; Sampaio et al., 2021) or hospital environments (Sugarman et al., 2021), limiting the generalizability of the findings to other contexts. Lastly, some samples were limited to particular orientations, such as CBT (AlRasheed et al., 2022) or psychodynamic and psychoanalytic approaches (Aafjes van Doorn et al., 2022), thus missing potential differences across various theoretical frameworks. Barker and Barker (2022) stand out for their attempt to examine the experiences of school counselors with a focus on therapists’ evaluation of TH effectiveness and appropriateness. However, like other studies, their sample included mostly women (85%), and the quantitative nature of the study restricted the ability to capture the nuanced perspectives of therapists.

The current study aims to delve into the therapists’ experiences of rapidly transitioning to a TH modality within a university counseling context that emphasizes a short-term treatment model and utilizes a variety of theoretical orientations. By examining these experiences, we hope to understand how therapists with these specific concerns and population parameters employ telehealth as an approach. We also sought to gain a deeper understanding of the barriers therapists faced and the strategies they used to navigate the complexities of an online format. We hope to contribute to the growing body of research by translating therapists’ experiences during the pandemic into concrete practical lessons for future clinicians who use TH in their practice. Identifying these challenges and the methods clinicians use to adapt their practice in changing conditions can enable mental health professionals and organizations to develop and implement the best possible practices and strategies for facilitating quick and effective transitions in the future.

## Methods

### Research sample and procedure

During the early period of the pandemic—from March 2020 to June 2020—we interviewed 13 licensed psychologists or psychologists in training who provided individual therapy, group therapy, and crisis services as part of a university counseling center at a large Western university. This convenience sample was utilized to capture the experiences of professionals who were all part of the same transition to telehealth services during this unique time frame. Inclusion criteria for this study required all participants to 1) have experience providing both in-person and telehealth services and 2) be part of the transition from exclusively in-person therapy services to exclusively online/telehealth services between March 2020 and June 2020. The sample included two pre-doctoral psychology interns, three post-doctoral psychology residents, and seven licensed psychologists and one licensed social worker. The age range of the sample was between 26 and 58, with seven participants self-identifying as women and six participants identifying as men. Eleven of the participants self-identified as white, and two participants self-identified as having a mixed ethnic identity. Participants’ therapeutic orientation/style is described in detail as Domain 1 in the results section. Each participant gave informed consent and was interviewed with pre-determined questions regarding their experience of transitioning to a TH modality during the pandemic (see Appendix A), using appropriate follow-up questions to clarify the participants’ statements as needed. The interviews lasted between 42 minutes and 141 minutes. The interviews were recorded, transcribed, and de-identified following the protocol approved by the university’s Internal Review Board.

### Data analysis

Qualitative analysis was conducted using the Consensual Qualitative Research (CQR; Hill, 2011) method by a team of researchers (more details provided below). The auditors had experience delivering clinical services and performing research in university counseling settings.

To briefly summarize the analysis: our first phase included reading independently through all the interview transcriptions to identify broad domains within the text, discussing in smaller teams (three coders per team), discussing as a larger group (all six coders) until we reached a consensus for any and all discrepancies for the identified domains, and then finally subjecting these domains to an audit. The next phases included reading independently, then working in smaller teams and as a full group to reach a consensus on core ideas, identifying categories and subcategories within the broader domains (cross-analysis), and then conducting an audit again. We then tabulated participant comments within each domain, category, and subcategory, which the auditors then checked for accuracy. In the end, all transcripts and codes were reviewed by eight team members (all six 6-coders + both auditors). The final phase included identifying quotes within the transcripts that best exemplify the categories/subcategories that also underwent a final audit.

The domains, categories, and subcategories were tabulated using the descriptors recommended by the CQR framework. Namely, we considered categories saturated by 12–13 participants general categories, categories mentioned by more than half (i.e., 6–11) of the participants were labeled typical, and categories endorsed by less than half of the participants were labeled variant.

### The research and data analysis team

The research and data analysis included six undergraduate student research assistants who worked as the core coding teams and two licensed psychologists/university faculty who served as the auditors. One of the auditors, who was also the principal investigator, conducted all 13 interviews and transcribed all the data. Understanding that having the principal investigator performing multiple roles would introduce bias into the final analysis, we had opted to have a second auditor who did not perform any of the interviews or initial coding to provide an additional perspective. Additionally, the research team attempted to mitigate the biases of the principal investigator by allowing the coding teams to perform their duties with minimal interactions with the auditors until after they had submitted the codes for audit.

All members of the undergraduate coding team were trained by the principal investigator, who was trained in CQR during a CQR training conference in 2017. The principal investigator provided six hours of didactic training and had the research team read selected chapters of the CQR manual (Hill, 2011). None of the undergraduate team members had experience with qualitative research prior to this project. However, both auditors had some experience with qualitative research, with the principal investigator having completed three prior qualitative projects. As part of the training, pilot interviews were used to help establish initial domains, core ideas, and categories.

### Positionality statement

When considering the positionality of the researchers, the coders were all students studying psychology with a future goal to work in the mental health field. Some of the coders reported having some experience with in-person and/or telehealth therapy formats as clients and/or as clinicians. The auditors were both licensed psychologists who had experience delivering in-person and telehealth clinical services and performing qualitative/quantitative research in university counseling settings. When asked to reflect on some of the biases members of the analysis team may have, most of the data coders reported having believed that in-person therapy services were likely to be superior to telehealth services, with the remaining coders believing it would be equally effective. Additionally, one of the auditors expressed having a negative experience providing telehealth, while the other auditor reported having a positive/neutral experience with telehealth services. We acknowledge these aspects of the research team’s positionality may have affected the analysis process, which we attempted to control by having regular meetings to discuss the coders reactions to the interviews and by requiring all six members of the coding team come to an agreement before having the auditors examining and finalizing the codes.

## Results

Our qualitative analysis yielded 13 broad domains, given in Table 1, with categories and subcategories also listed within the table. Please note the tabulation tables for each category and relevant subcategories as we describe these findings with representative quotes from the participants. In the interest of space, while we go into greater detail for domains one through eight in this manuscript, we have uploaded a full recounting of all categorized data and made it available [https://osf.io/5wh74/?view_only=9f31998f663a4b349dc0c662681ed50f]

**Table 1.** Overview of Therapist Experiences During the Transition to Telehealth: Categories, Subcategories, and Frequencies.

Domain	Category (Level 1)	Count Level 1	Subcategory (Level 2)	Count Level 2	Subcategory (Level 3)	Count Level 3
*For reference in “Count” columns: CQR descriptors include G—General (12–13 participants), T—Typical (6–11), and V—Variant (1–5)*						
I. Theoretical orientation & therapist style	A. Orientation/style (prior to TH)	12 (G)	1. Psychodynamic	2 (V)		
			2. CBT (also cognitive therapy)	4 (V)		
			3. ACT	2 (V)		
			4. EFT/Experiential (also Gestalt)	2 (V)		
			5. Solution-focused therapy	1 (V)		
			6. Humanistic (Rogerian, person-oriented, non-directive)	3 (V)		
			7. Relational (also interpersonal)	7 (T)		
			8. Integration/Eclectic/mix (also Wampold/Imel contextual model)	9 (T)		
			9. Relational cultural therapy (RCT)	1 (V)		
			10. Feminist	1 (V)		
			11. Adlerian	1 (V)		
			12. Compassion focused therapy (CFT)	1 (V)		
			13. Existential	1 (V)		
	B. Common interventions (specific tools used, including more basic descriptions of what the session might look like)	13 (G)	1. Identifying problem areas	4 (V)	a) Setting goals	2 (V)
					b) battery of assessments (assessing ability to describe emotional states)	1 (V)
					c) No subcategory 3	2 (V)
			2. Psychoeducation	8 (T)	a) Coping skills learning	2 (V)
					b) Use of metaphors to explain things	3 (V)
					c) Exploring medication options	1 (V)
					d) White board exercises/sharing screen	1 (V)
					e) Exploring external support	1 (V)
					f) Concept explanation	2 (V)
					g) No subcategory 3	1 (V)
			3. Crisis planning	1 (V)		
			4. ACT and mindfulness-based interventions	4	a) Developing psychological flexibility	1 (V)
				(V)	b) ACT hexaflex (acceptance, here-and-now, values, committed action, self as context, diffusion)	4 (V)
					c) Mindfulness (noticing somatic cues, noticing emotions, self-awareness)	2 (V)
					d) Meditation	1 (V)
					e) No subcategory 3	1 (V)
			5. CBT interventions	8 (T)	a) Cognitive restructuring techniques (positive self-talk, challenging thought patterns)	2 (V)
					b) Empowerment (pointing out strengths, encouragement)	4 (V)
					c) Homework	1 (V)
					d) No subcategory 3	3 (V)
			6. Experiential, here-and-now	10 (T)	a) Transparency	1 (V)
					b) Self-disclosure	3 (V)
					c) Silence	2 (V)
					d) Attending to nonverbals	4 (V)
					e) Empty chair (emotion-focused/gestalt approach)	3 (V)
					f) Two chairs	1 (V)
					g) Doubling (psychodrama)	1 (V)
					h) Mirror exercise	1 (V)
					i) Here-and-now	3 (V)
			7. Emotional processing and regulation	6 (T)	a) Regulation skills	1 (V)
					b) Emotion processing	4 (V)
					c) Emotion activation	1 (V)
					d) Clearing space (based on EFT)	1 (V)
					e) Trauma processing work	2 (V)
					f) No subcategory 3	1 (V)
			8. Building alliance/therapeutic relationship (happening between therapist and client)	10 (T)	a) Reflective listening/reflections, empathetic conjecture	3 (V)
					b) Therapist=observer/passenger, client-driver (giving control over the session to client to set direction, pace, etc.)	5 (V)
					c) Egalitarian relationship (equal power)	2 (V)
					d) Empathy	3 (V)
					e) No subcategory 3	1 (V)
			9. Psychodynamic work, making unconscious conscious	1 (V)		
			10. Thinking about existential questions, meaning, identity, etc.	1 (V)		
			11. Interpersonal interventions	2	a) Relational images	1 (V)
				(V)	b) Exploring relational patters	1 (V)
II. Obstacles and Changes Created by the Transition to TH	A. Negative changes/obstacles	13 (G)	1. Lack of non-verbal cues	12 (G)		
			2. Misfit of theoretical orientation/intervention tools (i.e. had to choose more cognitive approach or could not use specific tools usually used in-person, i.e. empty chair)	10 (T)		
			3. Therapist’s engagement level (less emotion/more cognitive, using more/less non-verbal cues, less/more directive, less/more interactive, etc.)	8 (T)		
			4. Process interruption (i.e. changes in procedural protocols or tech issues that get in a way of therapeutic work)	10 (T)		
	B. Positive changes/obstacles (things that helped to improve in-session interaction/outcome)	6 (T)	1. Changes in theoretical orientation/intervention tools	5 (V)		
			2. Therapist’s engagement level	2 (V)		
			3. other	2 (V)		
	C. Neutral/no Change or neutral obstacles (different, but not necessarily making things better or worse)	12 (G)	1. Changes in theoretical orientation/intervention tools	7 (T)		
			2. Therapist’s engagement level/in-session interaction	11 (T)		
III. Perceived Therapy Relationship Online vs. In-Person	A. Existing clients vs. new clients	9 (T)	1. Better with existing clients (those met in person before TH)	8 (T)	a) Easier to continue an existing relationship than to build one	6 (T)
					b) More emotional connection with existing clients	1 (V)
					c) Harder to create the right “vibe” without the therapist’s nonverbals	1 (V)
					d) Harder to get to know a new client	2 (V)
					e) Easier to catch nonverbals and other cues from existing clients	1 (V)
			2. Same (just as good) or different level of connection (neither positive nor negative)	3	a) similar level of empathy from the therapist	2 (V)
				(V)	b) Connection is more cognitive or feels less emotionally connected	1 (V)
	B. General comparison of dynamics in-person vs. online	13 (G)	1. Positive (better than in-person)	5 (V)	a) The format helps some client and/or therapist feel more comfortable	3 (V)
					b) Still feel a high level of connection	2 (V)
					c) No subcategory 3	1 (V)
			2. Negative (worse than in-person)	12 (G)	a) Harder to develop relationships or feel connected over TH; or therapist had a harder time feeling empathy or picking up emotions in TH format	9 (T)
					b) Fewer nonverbal cues interfere with the connection	3 (V)
					c) Reduces therapist’s capacity	3 (V)
					d) Clients less willing to show emotion; or relationship is more cognitive than emotional	3 (V)
					e) Loss of social connection that comes naturally from being in-person (i.e. the “energy” of being in the same room with someone); & awkwardness of interaction due to format	9 (T)
					f) Less privacy for therapist and/or client	1 (V)
					g) The client feels less supported or connected	1 (V)
					h) Tech issues interfering with the connection b/n client and therapist	8 (T)
					e) No subcategory 3	2 (V)
			3. Same/neutral	10 (T)	a) “Vibe” continues strong, at least with existing clients OR clients and/or therapists felt no difference in connection level	1 (V)
					b) Seeing a therapist represented normalcy for clients during the pandemic	7 (T)
					c) Just as open/expressive in this format	1 (V)
					d) Therapist felt similar amount of empathy	3 (V)
					e) Needed to change some things, but effectiveness was still there	2 (V)
					f) No subcategory 3	1 (V)
IV. Perceived Match/Mismatch for the TH Format	A. Therapist’s match/mismatch (therapist’s ability to provide service via TH)	3 (V)	1. Personal circumstances misfit (unique experiences affected the ability to be successful, i.e., ADHD, pregnancy)	3 (V)		
			2. Professional misfit (misfit of orientation, tools, etc.)	2 (V)		
	B. Client’s match/mismatch	13 (G)	1. Readiness (willingness to work online and in general, their commitment to work)	5 (V)	a) Match	3 (V)
					b) Mismatch	4 (V)
			2. Client’s diagnosis or presenting concerns	13 (G)	a) Match	7 (T)
					b) Mismatch	11 (T)
			3. Circumstances (long commute, low SES, busy schedule, availability)	10 (T)	a) Match	5 (V)
					b) Mismatch	4 (V)
					c) Neutral	1 (V)
V. Ethics and Boundaries Concerns Related to TH	A. Privacy (ability to withhold information from others. Ex: client not having a private space for therapy and worrying that family will overhear them is a privacy concern)	13 (G)	1. Client’s privacy concerns	13 (G)		
			2. Therapist’s privacy concerns (unintentionally disclosure of therapist’s private information, i.e., personal cell#, private space, etc.)	4 (V)		
	B. Confidentiality (refers to therapist’s need to keep protected information safe. The therapist not having a private space for therapy (where therapists kids of spouses can overhear on conversation) is a confidentiality concern.)	8 (T)				
	C. Client’s safety (self-harm concerns-difficulties handling crisis situations)	12 (G)				
	D. Boundaries	12 (G)	1. Work/life separation (not mixing working and life schedules, not answering after hours, not doing personal stuff during work time; and this can probably cover situations when family interferes into therapist’s work session?)	10 (T)		
			2. Communication rules (email is for scheduling not asking therapy questions or friendly chatting)	6 (T)		
			3. Setting in-session rules (addressing “too casual”/naked client situations, blurred lines b/n professional and personal (irrelevant chit-chatting), requiring proper location/privacy/internet connection, etc.)	5 (V)		
	E. Therapist competency in TH format (therapist’s concerns whether it’s ethical for them to do online therapy without proper skills/knowledge)	7 (T)				
	F. Therapy efficacy in this new format (is it ethical for clients with certain conditions not have in-person service available)	9 (T)				
	G. Legal issues / protocol compliance	10	1. Client’s location (out-of-state)	4 (V)		
		(T)	2. New protocols compliance (checklist of actions/docs: informed consent, emergency contact on file, safe location, etc.)	10 (T)		
	H. No concerns	1 (V)				
VI. Training and Protocols	A. Had prior training (official training before	2 (V)	1. Didn’t take seriously (not effective, wasn’t as helpful)	1 (V)		
	COVID)		2. Lack of training (had some but wasn’t enough when COVID happened)	2 (V)		
	B. Crash course / personal research into TH (once pandemic happened)	5 (V)	1. Provided by APA or other organizations	3 (V)		
			2. Personal research into the topic	5 (V)		
	C. Desired/needed training (once pandemic happened)	11	1. Preference for in-person training	2 (V)		
		(T)	2. Condition-specific interventions over TH (crises interventions + TH with trauma patients)	3 (V)		
			3. General training/info/knowledge (how to do the same things therapists did in-person but online)	6 (T)	a) General TH training	3 (V)
					b) Theoretical orientation-specific training	2 (V)
					c) Working with groups/couples over TH	2 (V)
					d) More data on TH and clients’ experiences with TH	2 (V)
			4. Learning about other TH therapists’ experiences (discussion groups, TH research results)	8 (T)		
			5. Clear new protocols	8 (T)		
			6. More training/time before starting work with clients (or more time to learn all new info & protocols)	3 (V)		
			7. TH supervision	2 (V)		
			8. Tech/platform training	2 (V)		
			9. Grad school training	2 (V)		
			10. Non-verbals training	1 (V)		
			11. Practice group	1 (V)		
	D. No prior training	2 (V)				
	E. Training seen as unnecessary/not needed (i.e. if they are already good at some aspect like technology use)	1 (V)				
VII. Logistics in the transition to TH	A. Transition to tech/software and all the tech issues/software misfits	12 (G)	1. negative	11 (T)	a) Connection disruption/poor connection	10 (T)
					b) Lack of equipment	4 (V)
					c) Poor equipment or software functionality (glitchy, etc.)	7 (T)
					d) Increased distractions	4 (V)
					e) Steep learning curve	3 (V)
					f) Time-consuming	3 (V)
					g) Needing to help others figure out the tech	4 (V)
					h) No subcategory 3	1 (V)
			2. Positive	8 (T)	a) Good equipment or software functionality	3 (V)
					b) Easy transition to tech	5 (V)
					c) Eventually, it went smoothly/was successful	1 (V)
			3. Neutral	1 (V)	a) Pros and cons end up about the same in the end	1 (V)
	B. Scheduling/ communication	13 (G)	1. Adjustment/discomfort with new protocols	1 (V)		
			2. Setting up appointments	8 (T)		
			3. Communication with a client outside therapy	11 (T)		
	C. Administrative (paperwork)/protocol/procedural	12 (G)	1. New checklist/procedural items	8 (T)		
			2. Legal & paperwork	8 (T)		
			3. Calls to CPS	1 (V)		
			4. Figuring out new procedures w/co-workers	3 (V)		
			5. Licensing requirements	1 (V)		
			6. Researching the new software	1 (V)		
			7. Deciding on safety protocols	2 (V)		
	D. Desired protocol/ actions (looking back, advice on how it could’ve/ should’ve been done better, specific to therapy training, logistics, and administrative transition protocols, for “our” center and recommendations to others)	8 (T)	1. Wanted advice from others experienced in TH	3 (V)		
			2. Wanted more knowledge or different choices made about software options	3 (V)		
			3. Desired more productive meetings/decision-making/leadership at the center	4 (V)		
			4. Wanted crisis response plan	2 (V)		
			5. Wanted regular consultations with colleagues about the transition	2 (V)		
			6. Wanted more preparation for dealing with boundaries issues	1 (V)		
VIII. Therapist’s Expectations About TH	A. Emotions related to TH (fearful, nervous, powerless, empowered, confident, feeling unprepared (in terms of training), etc.)	13 (G)	1. Before starting TH	12 (G)	a) Positive	1 (V)
					b) Negative	12 (G)
					c) Neutral or both	2 (V)
			2. When first starting TH	7 (T)	a) Positive	1 (V)
					b) Negative	6 (T)
			3. After starting TH	11 (T)	a) Positive	11 (T)
					b) Negative	7 (T)
	B. Evaluation of/cognitive beliefs about TH (effective or not; ‘like it’ vs ‘don’t like it’, will continue offering TH vs. never want to do it again)	13 (G)	1. Before TH	12 (G)	a) Positive beliefs (effective)	4 (V)
					b) Negative beliefs (not effective)	10 (T)
					c) Neutral	2 (V)
			2. After TH	13 (G)	a) Positive beliefs (effective)	12 (G)
					b) Negative beliefs (not effective)	11 (T)
					c) Neutral	10 (T)
	C. Energy levels related to TH format	13 (G)	1. Uses more energy	11 (T)		
			2. Uses less energy	1 (V)		
			3. Same energy (both or neither/just different)	5 (V)		
**OTHER DOMAINS**						
IX. Unique therapist circumstances (i.e. therapist has ADHD, or pregnancy, etc.)			A. Pregnancy/ maternity leave during transition (1-V), B. Mental condition (diagnoses, concerns) (6-T), C. Home office concerns (6-T), D. Therapist in therapy (1-V), E. Workload (4-V)			
X. Location of providing therapy effects			A. Effect on therapist (in office vs remote) (13-G), B. Effect on client (in office vs remote) (10-T), Effect on therapy (in office vs remote) (11-T).			
XI. Effects of TH format on client, their behavior & in-session interactions after transition.			A. Client’s engagement level (more/less cognitive/emotional, willingness to emote, feeling more/less connected to therapist, etc.) (13-G), B. Client’s attitudes, opinions, & preferences regarding TH (likes/dislikes, convenience, quit or continue, do they take it seriously) (10-T), C. Therapeutic progress (9-T).			
XII. Effects of pandemic (specifics mentioned in the interviews)			A. Therapists (10-T), B. Clients (4-V), C. Increased client-therapist communication when not in session (1-V), D. Concerns about physical health/ COVID illness (5-V), E. Challenges of “quarantining” with other household members (4-V), F. Uncertainty of planning ahead (3-V), G. Changes in logistics/ workload due to pandemic (2-V).			
XIII. Counseling modalities (fit and obstacles)			A. Impact on group therapy (7-T), B. Impact on couples therapy (3-V), C. Hypothetical cases (3-V).			

### Domain 1: theoretical orientation and therapist style

Before the transition to the TH format, our participants reported a range of theoretical orientations and styles, with nine out of 13 participants adopting an eclectic approach to therapy, integrating various orientations and tools. Additionally, over half of the participants (7/13) indicated using a relational or interpersonal approach with their clients. Two variant subsets showed, on the one hand (4), a preference for humanistic methods, including Rogerian, person-oriented, and non-directive approaches, and, on the other hand (4), directive strategies such as cognitive and cognitive-behavioral therapies (CBT).

Participants also reported using other specific orientations and theoretical approaches, such as psychodynamic (2), acceptance and commitment therapy (2), experiential and emotion-focused (2), solution-focused (1), relational cultural (1), feminist (1), Adlerian (1), existential (1), and compassion-focused therapies (1).

We also asked our participants about their preferences for in-person interventions prior to the transition. The data showed a typical trend towards experiential interventions (10/13), including emotion-focused techniques like two-chair (3), focus on here-and-now (3), attention to non-verbal cues (4), and use of self-disclosure (3).

Another typical subcategory among the participants was their emphasis on building an alliance, or therapeutic relationship, with a focus on what was happening between the therapist and the client (10). Within this subcategory, five participants referred to their clients as drivers who have control over the session, while they (therapists) are just the passengers; others emphasized the importance of reflective listening (3), empathy (3), and an egalitarian relationship (2).

The data also revealed other typical tendencies, such as the use of psychoeducation (8) and CBT tools (8), with a focus on empowerment (4) and cognitive restructuring techniques (2). Among variant trends, our data identified the use of emotional processing and regulation techniques (6) and acceptance- and mindfulness-based interventions (4).

### Domain 2: obstacles and changes created by the transition to TH

Our analysis revealed the emergence of negative changes and obstacles encountered during the transition from in-person therapy to the TH format, which was consistent and shared across all 13 participants. One major concern expressed by most participants centered around the substantial impact on their therapeutic approach—including their interventions and in-session interactions—attributed primarily to the limitations posed by the online format.

A recurrent sentiment was the heavy reliance by therapists on patients’ nonverbal communication in traditional in-person therapy, which in nonverbal discernment was significantly disrupted by the transition to TH. A general subcategory (12/13) referred to challenges associated with the restricted visual field in online sessions and thus the lack of access to non-verbal cues, as Participant 2 said, “I can only see maybe 10% of what’s happening. … I usually only see my clients from the chin up, sometimes, I’ll see shoulders, … so I only have facial cues. … And then there’s video lag. That happens quite a bit”.

Not having access to full-body language left therapists grappling with the perception of missing vital cues, making it “harder … to interpret the emotional experiences of [their] clients” (P6), resulting in a common sentiment of increased difficulty in achieving the same level of connection with their clients. For example, Participant 1 stated, “[N]ow I’m relying almost solely on their verbal communication. … I don’t think it’s possible to build a rapport as quickly. … I am struggling more with empathy, based on the lack of nonverbal communication”.

In addition to being unable to catch onto clients’ non-verbal cues, Participant 8 highlighted the inability to leverage their own body language for client engagement. They described the challenge, noting the absence of physical cues like leaning forward or adjusting the therapist’s chair “to stop someone or to intervene”, and added that “talking to a client sometimes feels like … watching a movie. It doesn’t even feel like I’m interacting with someone”.

Our analysis revealed another typical trend within this category, with 10 out of 13 participants encountering difficulty maintaining consistency with their theoretical orientation or intervention tools after transitioning to TH. Some therapists found it necessary to discontinue certain intervention tools integral to their in-person practice. For instance, Participant 8 remarked that they “had to stop using some of [their] intervention tools like two chairs because doing them online was more difficult or ineffective”. Participant 7 echoed a similar sentiment, “It feels kind of weird to do [spatial interventions] over teletherapy”.

Furthermore, the struggles with implementing certain interventions in online settings pervasively impacted how therapists interacted with their clients, revealing another typical pattern (8/13) characterized by a tendency to adopt more directive approaches. Participant 8 shared their experience:

When I first started doing teletherapy, I was more relying on CBT interventions … I was … being more directive than I typically like to be in therapy, which I wasn’t liking. … I think that leads into why I was doing more CBT or active interventions, is because I rely on a lot of those nonverbal cues to do relational interventions.

This shift in therapy style was marked by increased loquacity and a transition towards being “more cognitive and less affect-y [sic]” (P2). One of the participants (P1) shared that they noticed themselves “giving more advice… because empathy is more difficult [in an online format]”. While some therapists expressed discomfort with silence during online therapy, as opposed to in-person sessions, leading them to adopt a more directive approach and feeling “the need to carry the session” (P8), others (P11) recognized the value of silence as an opportunity to concentrate on the limited audio and video information. Participant 4 mentioned using silence to “give [clients] the space so that [the therapist] can make sure [they] …understand what’s going on”.

The TH process itself became a challenge—and sometimes an impediment—as we found in another typical subcategory (10/13). Adhering to new TH protocols consumed valuable time that could have been dedicated to meaningful therapeutic conversations addressing clients’ concerns (P1). Technical issues, such as loss of or poor internet connection and the need to troubleshoot during sessions, not only evoked frustration in therapists and clients (P7) but also hindered therapeutic progress. These challenges interrupted their empathic connection, fostering a sense of disconnection and “disjointedness” with the client (as recounted by Participant 11), occasionally resulting in the loss of crucial moments in the therapeutic healing process. As noted by Participant 1:

The client was just getting into an emotional place, talking about some relationship issues, and I was following along with them and then, blip, black screen. … It took like 5 minutes for my power to come back on and … like 5 minutes more before I was back online. … We were moving into some really emotional content that was the reason the client was coming in, and then black screen. That was a really critical period that was missed.

Our participants also noted positive experiences, with six of them giving examples of improvements in their therapy style and in-session interactions. For instance, Participant 9 reflected on their experience of asking more questions during sessions and recognized the importance of teaching clients to consistently check in with themselves outside of therapy. Another participant (P13) highlighted a positive example exclusive to the TH format when the video camera perspective helped the therapist to observe and address interpersonal behavior related to height concerns between a client and their partner:

Interestingly, the control over the video cam perspective on the client took on a depth of height in relation to me. That was something I was able to notice and point out as it related to exactly the concern this client was expressing. And that proved to be useful and was something that came directly from doing the teletherapy format that we wouldn’t have gotten to when we’re face to face.

One more participant (P11) shared a case when the TH format “gave [them] a little bit more therapeutic courage” to engage in a powerful, emotionally focused intervention early in the treatment, that they otherwise “would have been too afraid to do” at that moment in a face-to-face session. The perceived safety of the online setting helped this therapist feel confident in adopting a more direct approach, which ultimately shifted the course of therapy and strengthened the couple’s relationship.

This study showed that despite abrupt changes, participants were generally able to adapt, and therapy remained fundamentally consistent. Twelve out of 13 participants noted that the adjustments they had to make were neither bad nor good. For instance, Participant 13 admitted that they were “not sure there’s been too many noticeable changes”. They added, “I still felt like I was asking the same kinds of questions, making the same kinds of statements, intervening at some of the same points that I would otherwise”. Another participant (P4) shared:

I think I sort of had an expectation that it was going to feel quite different. I don’t know if you want me to quantify that, like 50% different. … I feel like in reality, it’s been maybe 20% or something like that, like 20 to 15%: pretty minor modifications or things that didn’t feel as major to me as I was anticipating, I guess.

Change in the manner of engagement between therapists and clients was a typical subcategory (11/13). Participant 4 gave an example of having to be “a little more explicit and sharing that I was having an emotional reaction with the client” in case the client did not have a good visual of non-verbal cues from the therapist. Participant 5 shared that they tended to “check in with [their] clients more through teletherapy than in face-to-face. … [For example,] how are you doing? What are you feeling? What’s coming up for you? Again, more of that kind of explicit”.

Regarding potential changes to their overall therapy style, seven out of 13 participants agreed that while they had to make some changes, their overall style and interventions remained the same. Participant 1 emphasized, “The way I still conceptualize cases and treatment planning is still the same”. Participant 3 affirmed, “I don’t think it has greatly changed my style. I take pretty seriously the idea that the most effective tool you have in the therapy room is you and what you bring to it. I mean, I’m still me, right?”

### Domain 3: perceived therapy relationship online vs. in-person

The changes in the perceived relationship between therapist and client were viewed as generally negative among our participants (12/13) following the transition to TH. Nine participants said they found it more challenging to feel connected with their clients online. Participant 2 admitted, “It’s harder to feel as much empathy like normally in person”. Participant 11 noted that they felt the same level of connection with the client once they got to an emotional place, but otherwise, it was “harder to stay in the moment with the client throughout the session”. They noted that the presence of the screen created a barrier to connection, stating, “It’s almost like I’m not interacting with a person as much as I’m interacting with the screen”, and that they had to remind themselves that there was a real person in front of them.

A typical finding among participants (9/13) was the expression of concern that they were missing the natural connection that comes from meeting in-person. These therapists stated that “there is something essential [about the in-person experience] that is just missing” (P6) and that what was missing could not be totally bridged in the TH format (P12). Participant 2 emphasized the dynamics of face-to-face interaction, stating, “I get a lot of energy from in-person interaction … I can feel where they are at, I can feel where I’m at. It’s more dynamic, my brain is more engaged, every part of me is more engaged”. Other participants noted that the unique energy and connection present in a face-to-face setting facilitated a sense of control and safety that, in turn, contributed to feeling more comfortable (P9) and “increased the experience of intimacy emotionally just by being there” (P13).

The loss of this unique connection from meeting in-person raised concerns among therapists about the potential negative impact of the change on therapy outcomes. Participant 1 expressed a preference for in-person sessions, believing that it would help some of their clients “feel more connected … like some of those feelings of being cared about might be a little bit stronger if we were in the same room”. Participant 11 also shared a case example where they believed they would have been able to sustain more emotional sessions and “do deeper level work that would have been beneficial to [the client] if we were still working in the office. I feel like with that client something was lost that we just were not going to get back”. Participant 4 added that while they could still use the same therapeutic skills and tools online, the feeling of having “another person here in the room with you who cares about you” was missing, impacting the support clients felt.

Three participants felt the new TH format reduced their professional ability to connect with clients. For instance, Participant 1 stated, “I want to be at 100% of my interpersonal skills, and I feel like [the TH format] handicaps me down to 20%”. Participant 2 noted that their emotional connection was only “60–70% on the best days”, while Participant 4 quantified their level of connection as “probably like 85%”.

Even though almost all participants but one highlighted negative aspects of the changes to the relationship, the data showed a typical, contrasting trend: 10 out of 13 participants assessed the overall perceived relationships with their clients as unchanged or just as good. Seven therapists in this subcategory reported that the connection levels remained strong. Some participants (P9) indicated that they had not felt any real shift, and (P13) still felt just as connected as ever. Participant 5 noted that while they felt that “effectiveness was diminished, they still were connecting well and getting work done”. Three participants also mentioned not noticing a huge difference in clients’ levels of openness or emotional expression (as indicated by Participant 4) or their ability to form and build relationships (as indicated by Participant 7).

Interestingly, five out of 13 participants reported positive experiences about their perceived relationships with clients after switching to TH. Participant 7 admitted to being “quite surprised by how connected [they] have been able to feel with the clients and how productive the work felt in a lot of ways”. Participant 12 stated they had “lots of positive experiences as far as feeling really emotionally connected to the clients and them feeling really emotionally connected to [the therapist]”.

In contrast to the earlier stated fears about a worse connection leading to negative outcomes, two therapists found that conducting therapy sessions online led to more progress with certain clients. Participant 4 reported that a client with gender dysphoria felt “more comfortable to share” in the TH format. At the same time, another male therapist explained that having the distance in the digital format helped to build trust and connection with his client, who had a significant trauma history and an aversion to men (P6). However, this therapist also noted that while they were more connected in this format, he was unsure if this connection would meet his client’s long-term treatment needs.

Comparing their experiences of TH with clients they had previously met in-person versus clients they had never met face-to-face, nine participants spoke about this contrast. Among the typical trends observed was that they perceived a better therapy relationship with clients they had worked with in-person before the transition (8/13). Six of these participants highlighted that it was easier to maintain an existing relationship than to build a new one. Participant 6 articulated this sentiment, stating:

If this is an individual client who I saw pre-COVID or pre-teletherapy, I don’t think the connection has changed at all. I think it’s been able to maintain its course and even become stronger over the course of time. If I’m meeting with a new client, who we’ve only been doing teletherapy exclusively, I feel like I have a harder time making a connection with them. I don’t think it’s detrimental to the therapy, but I notice I just don’t feel as close to them as I do with my in-person clients.

Similarly, Participant 1 reported that their clients who have been exclusively TH clients were less willing to emote, making it harder to establish rapport. Participant 3 admitted that they did not know their new TH clients as well as their in-office clients. Participant 9 noted the advantage of having already established connections, “so even if there’s something that goes really weird, there’s already a good rapport there”.

When making the same comparison, three participants reported the same levels of connectedness with their clients regardless of whether they had met them in-person before. Participant 13 explained, “I feel as connected with some of the clients that I have not seen in any other context other than teletherapy, and I feel just as connected with some of the clients that I had seen previously”. Participant 4 admitted that while they never felt as connected as they did in person, they were surprised by how connected they felt online. Meanwhile, Participant 2 emphasized that the perceived therapy relationship with new online clients was “just a different connection style”, neither better nor worse.

### Domain 4: perceived match/mismatch for the TH format

We identified a general concern among participants regarding the sustainability or “match” of clients and therapists for TH, with all 13 participants focusing on the clients’ goodness of fit. Our data showed client diagnosis and presenting concerns were typical factors for match/mismatch classification (11/13). Participants mentioned the following concerns: clients experiencing active suicidality (8/13), manic or psychotic states (4), severe substance use disorders (2), trauma (1), and borderline personality features (1).

Participant 1 shared their worries about clients in active crisis, stating:

I’m also thinking in the back of my mind, ‘What if I can’t do this? They are so far away. How do I? You know, what’s my next step that I have to do? I have to—if this call ends—I have to call … the police.

Another participant (P4) echoed similar concern about not being physically present with someone at risk of self-harm, not being able “to try to take action to keep them safe, [and thus leaving them] in a dangerous spot as far as suicidality”. Participant 3 emphasized that for the “actively psychotic, you should be in the hospital and not in teletherapy”.

To contrast these perspectives, several therapists mentioned that even if some clients were not the best match for online therapy, clients with the most challenging concerns could and should still be seen. One therapist (P6) suggested, “There are some extra steps you have to take for more risky or suicidal clients, but I don’t think that should exclude them from receiving these types of services”. Another (P3) said, “I have such a hard time with it because I feel like we’re ultimately saying … the people who need it the most are not appropriate for it and can’t get help”. Participant 9 concurred, stating, “Offering something is better than nothing”.

Our data showed seven participants recognized the TH format as a good fit for some clients; however, there was some disagreement about which diagnoses made a client a better match for online therapy. For instance, while Participant 2 felt that clients needing “cognitive worldview processing” rather than trauma work were a better fit, Participant 4 felt that trauma was not a mismatch, noting:

I actually have worked with some trauma clients … in the limited experience that I’ve had, I feel like most of my clients have been a pretty good fit for teletherapy. I haven’t had a single one that I’ve said, “OK, I think we’re going to have to figure something else out”.

Another diagnosis with differing views about fit for TH was autism. Participant 9 felt that clients on the autism spectrum might do better with this format “because it’s not the direct eye contact, so it feels less threatening”. In contrast, Participant 8 felt that working with a particular client on the autism spectrum was more difficult in online setting, explaining, “She makes no eye contact. Super, super hard to do teletherapy with … someone with … those social difficulties or … lack of social awareness. It’s easier when in a physical presence”. Citing another diagnosis, Participant 9 believed that agoraphobia was a good match for TH because of the initial higher comfort level:

This [format] is probably great [for them]. Is it going to limit their ability to have exposure? Potentially? But you could also work with them if they have a tablet or a phone to say, “OK, I can be with you while you step outside your house, you know, and let’s do that exposure”. So, [TH], I think, could actually be a really nice format.

Citing certain client circumstances as a factor for deciding a match/mismatch for TH proved to be a typical response for participants (10). Four participants stated that the TH format was a better match for some clients due to its convenience. For instance, Participant 2 noted that some of their clients might not have made it to therapy if it had to be in-person due to circumstances, “like students who have a long commute or really low SES and have other barriers to coming in for treatment”. Others agreed that TH was a good—if not better—alternative for clients from rural communities who “would need to travel long distances or have transportation issues” (P9), people with “super busy schedules” (P4), and “people coming in on their days off of school and having to take work off and all of that versus being able to just kind of log into an appointment” (P10).

Poor fit due to client circumstances was another variant topic addressed by participants (4). For example, Participant 5 highlighted that some clients might “have difficulty accessing technology in general due to their socioeconomic status” (i.e., unhoused people), while others might not have private space to do the therapy without being overheard by family members or roommates. Other therapists in this group indicated that clients had to have access to proper technology and a stable internet connection to do TH (P7); “if they don’t, it’s a deal breaker” (P11).

A variant subcategory included client readiness for therapy, with five therapists discussing factors such as a client’s willingness and commitment to work online. Participant 1 shared the challenges of TH with clients resistant to treatment, stating, “I can’t provide therapy if you’re not willing to meet me where we need to meet”. Participant 10 emphasized that TH “can be effective as long as both parties are into it”. Participant 11 described a couple that was not a good match for TH because of domestic violence and other “red flags … that made me realize I need to be able to see you, not just hear you”. They summed it up, “I think it depends on the client. It depends on the case. I don’t think there’s a one-size-fits-all kind of thing for [TH]”.

While most of our participants were concerned about a good match between the format and the client, three expressed worries about themselves as therapists being a poorer fit for TH. Participant 1 felt that their ADHD diagnosis made it more difficult to focus during online interactions, noting:

I also identify as having been diagnosed with ADHD, and over the phone that I’m sitting here, you know, playing with stuff, finding stuff on my desk, resisting the urge to play with stuff … and then re-focusing my attention on things. … I’ve learned about myself … that I’m not a multitasker. And so, I do well with individual therapy with somebody right across from me. I can focus on what they’re talking about, but [online] there’s an aspect of that that feels like multi-tasking to me.

The other two participants with statements in this category also reported difficulty remaining present during online sessions without losing the train of thought (as indicated by Participant 2). As noted by Participant 11, the difficulty concentrating had a lot to do with not interacting with a person as much as interacting with a screen.

### Domain 5: ethics and boundaries concerns related to TH

Our analysis revealed another general domain: the transition to TH raised significant ethics and boundaries concerns among all 13 participants. These concerns included privacy (13) and confidentiality (8), client safety (12), professional and work-life boundaries (12), therapist competency (7), therapy efficacy (9), and legal issues (10).

All 13 therapists expressed their worries about privacy in TH, such as clients not having a private, safe space for therapy sessions. Participant 8 gave an example of a client describing this issue, “I worry about my family overhearing, and I just really don’t feel comfortable just doing therapy here in my home”. Participant 5 emphasized that some of their clients were homeless and “finding spaces where they can dwell is really difficult and challenging for them”. Another participant (P2) shared a concern about losing control over privacy in an online setting, “People’s confidentiality … I don’t think sometimes clients just aren’t very cognizant of that … I can’t manage any of that, … I can’t protect their confidentiality there. It’s their job. All I can do is … mention my concerns”.

Participant 11 mentioned group therapy with concerns around privacy and confidentiality, saying, “It’s no longer my sole responsibility—the facilitator’s sole responsibility—to maintain confidentiality for the group. Now it’s everybody’s responsibility”. Therapists themselves also faced privacy concerns in the TH format. Four participants worried about the risk of unintentionally disclosing personal information during online sessions, such as their cell phone number or elements of their private space. For instance, Participant 2 explained:

There are things that I don’t normally share … in the therapeutic space. … Usually, it’s a choice for me to like say, “oh yeah, I have a dog”. But now it’s not really a choice. Now it’s like I have to say I have a dog [in case it barks]. … So, there are things about my life that I don’t have a choice about anymore.

A variant category in this domain concerned the challenges therapists faced to ensure confidentiality (8/13). When it comes to maintaining confidentiality on the therapist’s end, Participant 3 noted, “I’m definitely more concerned with confidentiality … just because of where I’m doing therapy. And I mean I put a sign on the door, I tell people not to come in”. Another participant (P4) provided an example where their spouse interrupted a session due to an emergency involving their child choking. One more participant (P6) brought attention to the challenges of maintaining client confidentiality when working from home in a small house shared by others: their wife had to sit in the living room topless after their baby threw up on her during a session, as there was no private space available to address the situation without compromising confidentiality.

Regarding maintaining confidentiality on clients’ end, Participant 10 discussed this saying:

Back to the kind of general piece of confidentiality, I don’t have much control over the environment. I don’t know if someone’s husband or friend or something that’s going to walk in. … I personally can keep your information secure, but I can’t ensure it all around.

Participant 12 added, “I feel little divided on that, because in some ways it feels like people ought to be responsible for their own confidentiality”. This raised the important question of whether it is the therapist’s or client’s responsibility to ensure the client’s space is confidential, beyond the therapist’s duty to warn and explain potential issues of not being in a private setting.

Another general concern—raised by 12 participants—related to client safety and the difficulties in managing crises remotely because of therapists’ lack of control over the client’s immediate environment. Participant 9 stated:

Something goes awry, so this person needs to be hospitalized. I’m going to be feeling a whole lot better when I’m in that room. … Versus someone goes “click” like, “Oh crap, what just happened”. … so, I think there is that sense of a little bit more of that control/safety in that sense that gets created by being in person.

Issues with maintaining professional boundaries in TH presented another general category in this domain (12/13). Many participants described challenges separating work from personal life, such as not mixing work and life schedules and not answering client messages after hours (10). For example, Participant 5 shared that in their attempt to be available for their clients during such a critical time, “I didn’t really draw any firm boundaries for a little while, and it was … messy and blurry. And I was talking to clients everywhere from 7 AM … till 10 o’clock at night”.

Six participants emphasized setting communication rules for themselves and their clients, such as using email primarily for scheduling rather than therapy discussions or casual conversation. Participant 2 shared an example where their client sent daily videos of their emotional support cat, prompting a discussion about establishing boundaries and limiting messaging to scheduling purposes only.

Another variant subset included five therapists who reported having to set certain in-session rules to address issues like clients being too casual, wearing inappropriate attire, or having sessions in unsuitable locations such as in the drive-through. One participant (P2) reported a client who joined the online session while being shirtless in bed, “the boundary of … me being in like his personal … resting/sexual space … it was just gone. … I don’t know where is the line? or … how do you know when you’re getting into … weird waters?” Another participant (P3) talked about a client with a 5-year-old daughter who kept entering the room and interrupting the session, “I have a hard time because, in the office, it would be really easy for me to be like, ‘hey, … we really can’t have your kiddo here. … But that’s just not where we are right now in the world”.

Legal complexities were a core category in this domain (10/13). The participants highlighted the importance of having a checklist of new legal and procedural actions to ensure compliance with state laws and licensing requirements and to provide safe and effective care. Participant 3 summarized their concerns:

Most of my discomfort was just around like am I doing all the things I need to be doing? Am I getting all the information I need to be getting to make sure that this is safe, legal, and ethical for my clients and for the center?

Additionally, four participants expressed concerns about doing therapy across state lines. Participant 6 reported, “I’ve run into some issues about clients going over state lines and whether or not I’m allowed to treat them in this format”.

Participants reported issues regarding therapist competency in TH (7) and the overall efficacy of TH (9). Seven participants questioned whether providing TH without proper skills and knowledge in this new format was ethical. Participant 1 admitted, “I think the biggest, just ethical concern is, am I qualified to do this … Even my lack of or my decrease in empathy seems unethical”.

Nine participants questioned the efficacy of TH, wondering about the ethical considerations of using it for clients who might benefit more from in-person interactions. Referring to the question of match/mismatch, Participant 3 stated that, in general, if the client was stable for outpatient therapy (vs. inpatient care), they were also appropriate for TH. Another participant (P9) reflected, “I think it goes back into that argument, is something better than nothing? … I tend to be one who probably said something is better than nothing”.

In contrast to ethical concerns, many participants stated their overall view that they can continue to practice ethically. As Participant 4 mentioned, “I think I had some preconceptions of clients that I didn’t think it would be good for teletherapy, and that’s changed with experience… things like anxiety, depression, eating and body image concerns, trauma… it’s gone relatively well”.

Although Participant 7 initially commented on confidentiality issues, later in the interview, they said:

I don’t feel like there’s really that many ethical things…. I’ve been kind of surprised… then looking at the ethics training it all just felt like basic stuff to me. Something like this doesn’t feel like that big a deal I guess… it ends up being more just procedural stuff, that’s like doesn’t feel very ethically ambiguous.

This reflection highlights the disparity between initial apprehensions about ethical complexities and the actual experience after getting past procedural adjustments. Participant 11 emphasized the importance of preparation, underscoring the need for thorough training to navigate the ethical and practical complexities of TH:

Before you start doing this, do your homework first. … Know what you’re getting into. Educate yourself. First, know about the different platforms. Know the ethical and legal responsibilities. Have an informed consent document in place. Go to those webinars. … know the laws first. Have a plan, … and then at the end of the timeline, that’s when you should actually start doing the work.

### Domain 6: training and protocols

A lack of preparedness to provide TH was common among our participants. Only two participants had received official training prior to the COVID-19 pandemic, but even they noted that the training they had was inadequate (P1), and they still “felt ill-prepared” to switch to a new format (P5).

The training available to them at the beginning of the pandemic was mentioned by five of 13 participants, as they were situationally forced to transition to TH and put their efforts into preparing to provide therapy online. Participant 3 admitted, “I had zero training before the fact, and it has been a very steep learning curve”. Participant 5 shared a similar experience:

Initially everyone was kind of like deer in the headlights, like, what do we do? And we couldn’t quite figure it out, so everyone went and did their own studying … I’m not going to get the answers in the time that I need, so I kind of took it on myself to just dig around and see what people were doing and what was ethical.

To prepare for TH, our participants read various articles and books, watched videos, attended webinars, and studied other training materials and guidelines issued and/or shared by professional organizations. Participant 5 noted that these materials were not the most comprehensive and did not address every issue, but they were helpful. Participant 12 added that while they could have used more training, they felt confident and competent to provide TH.

Despite some participants reporting confidence in their initial TH training at the moment of transition, most therapists in our sample (11/13) expressed a strong need for additional training once they had made the switch. Eight participants said learning from other therapists with TH experience would have greatly benefited their own transition. Participant 2 detailed this, saying:

I would have loved … to hear from people who practice similar types of therapy as me [who have] already been doing teletherapy and how they manage it. And since starting this … I staffed with other people in my cohort who are across the country doing teletherapy … seeing how they’re handling it, and [their] experience has been pretty similar to mine. … [I’m] trying to … converse with people, seeing if there’s any other ways that I can be managing things.

This desire for guidance from experienced teletherapists was echoed by Participant 3, who highlighted, “I think that is what I would have wanted probably the most is … talking to someone who has done this, who has experience with this, and in our center that doesn’t exist”. Another participant (P5) shared a similar sentiment, wishing they had had access to existing findings on TH and its outcomes to understand “what kinds of things create a more effective teletherapy experience … and then using that [information] as … foundation and guide for going forward”.

Two participants emphasized the helpfulness of staff meetings. During these meetings, therapists could raise various issues they had encountered, discuss them as a group, share perspectives, and troubleshoot to find solutions. Participant 8 stated, “In staff meetings, people are bringing up these issues and their problems with it. And then we can kind of troubleshoot or I can have awareness … that [something like what was shared] might happen with one of my clients”.

Further analysis of the clinicians’ desire for TH training revealed a typical need for ethics guidance, including clearer protocols for confidentiality, privacy, and TH procedures (8/13). Therapists in this subcategory shared a strong concern about whether they were legally doing everything possible to create a safe and ethical environment for their clients. For instance, Participant 11 admitted that they “would have loved just like a four-hour or just like a half-day workshop … just covering various topics … the ethical concerns [therapists] need to look out for [in] TH”.

Participant 13 added that there were not many guidelines at the time of transition, explaining, “The most difficult parts were just ensuring the professional and ethical care and standards were being followed. The most challenging part was because there weren’t a whole lot of standards and guidelines at the time”. Participant 3 shared their discomfort, questioning, “Am I doing all the things I need to be doing? Am I getting all the information I need to be getting to make sure that this is safe, legal, and ethical for my clients and for the center?”

Additionally, our data revealed some variant demands for more training in particular areas: group and couple therapy modalities (2/13), different theoretical orientations (2), condition-specific interventions in the online format (3), specific technology and platforms (2), and TH supervision (2).

### Domain 7: logistics in the transition to TH

Logistical concerns about the transition to TH fell into four main categories: technology and software issues (12/13), scheduling and communication problems (13), administrative procedure or protocol (12), and desired protocols or actions (8).

Most comments about technology and software were negative, with the largest being about poor or disrupted connections (11). Participant 2 estimated that “probably about 20% of the time” there were technological problems, stating:

Maybe the audio isn’t working or I’m having a video lag, or … we have to switch to a phone call, or … they have to move to a different part of their house, or they have to plug in a different device or whatever. It’s just …such a pain.

Participant 1 summarized the frustration:

If they cut out in the middle of the session, it really interrupts any empathic connection that has been established that session. … We’re crying and then wait, I can’t understand you, I can’t understand what you said. I can’t hear you. Then instead of them staying with their emotion there, [we’re] like “Can you hear me now? Are we connected again?” So. that inconsistency is really difficult.

Another participant (P6) shared an experience working with an emotionally closed-off client when technological interruptions disrupted therapeutic progress:

And this one session, she finally started opening up and exploring these emotions. And once she got to that point, our internet started cutting out and there was this huge lag time [when] she would cut out. I would cut out and …I just wasn’t able to do the work that I needed to do [be]cause I didn’t have enough information and we had to restart the call. We ended up finishing the call via telephone [be]cause the connection was so poor, but just due to that two-minute transition from teletherapy to phone, we lost that emotional experience, which was really disappointing for me.

The next-most mentioned problem concerned equipment or software problems, with seven people lamenting glitches in software or frustrations with equipment. Participant 11 related that the software for group therapy was particularly frustrating:

It was horrific when we were using [specific website 1] for group [therapy]. It was nightmarish. It was horrible … … What stands out to me the most is a group that I did during the end of the spring semester with [another therapist, and] … every session involved technological problems.

Other negatives mentioned in this subcategory included lack of equipment (4), increased distractions (4), the steep learning curve (3), the time-consuming nature of it (3), or needing assistance from others to figure out the technology (4).

On the other hand, seven mentioned positive experiences with the technology, including good software functionality (3) and feeling the transition was smooth (5). For example, Participant 7 reported, “I anticipated having a little more of a learning curve in terms of figuring out the software, but I feel like it was all so simple that it … was easier than I expected it to be”. Another (P13) noted, “As it’s turned out, it’s become a much more comfortable transition that I’ve actually enjoyed and appreciated”.

One aspect of the transition that made things difficult was adapting to new scheduling and communication protocols. Every participant mentioned challenges with this, including needing to adjust to new protocols (P1) and communicate with a client outside therapy (P11). Communicating the new TH system and setting up appointments was left to the individual therapist instead of the office staff. Eight therapists discussed needing to adapt to this. For instance, Participant 7 noted that they had to personally contact clients to inform them of the new procedures.

Under the category of administrative protocols, concerns clustered around the subcategories of new procedural items (8) and paperwork protocols (8). Among the new procedures, therapists had “to make sure [clients are] in the same location they have said they are at, that they’re alone in the room, that their emergency contact information is the same” (as indicated by Participant 1). Also, seven participants discussed difficulties with digitally signing all necessary paperwork, including intake, release of information forms, and informed consent for TH.

### Domain 8: therapist’s expectations about TH

The attitudes, opinions, beliefs, and expectations about TH, as held by our participants, were discussed at length (13/13). Therapists identified their emotional responses to and cognitive beliefs about TH, and the new demands of this format on their energy levels. The emotions ranged from fear and nervousness to empowerment and confidence, evolving as therapists gained more and more experience with the format. Before starting TH, only one therapist (P11) reported positive feelings, stating, “I felt eager, enthusiastic. … There was a little bit of excitement, and there was a little bit of nervousness”.

In contrast, the more general pattern (12/13) was characterized by negative emotions about the shift to TH, such as fear, nervousness, and discomfort about the transition, describing feeling unprepared and uncertain about what to do. Participant 1 recalled, “Feeling competent doing teletherapy was the most difficult part. I was very insecure about my ability to provide good therapy that’s not in person”. Another participant (P5) shared, “I would not have done this if I didn’t have to, because I was ignorant and I was afraid, you know, and maybe felt inadequate and some shame or something”. Participant 9 added, “Not at all comfortable, as far from comfortable as you can imagine”.

As time passed and therapists became accustomed to the new format, 11 therapists reported having more positive emotions toward TH. For instance, Participant 5 shared feeling “more comfortable and … satisfied that progress and work can still be done”. Another participant (P8) noted that they no longer felt nervous but “good about doing [TH]”.

In contrast, even with some passage of time, seven therapists continued to report experiencing unease and discomfort working in the online format. For example, Participant 5 admitted feeling “on edge and nervous”, particularly at the beginning of the sessions, but becoming more relaxed and comfortable as the session progressed. Another participant (P8) shared that they felt out of control when working with clients in distress, worrying about potential disconnection. This made them feel “helpless, … a little bit more powerless, and a little bit more worried, and … super nervous”. They added, “I don’t feel like I’m as good a therapist, just personally, maybe other people feel like they’re just as good a therapist, but I feel like I’m not as good a therapist”. Furthermore, one participant (P12) reported, “If anything, my discomfort has grown over time … and I think some of that actually for me was just novelty; …and so, the novelty… kind of focused my brain. Now the novel[ty] is gone, it’s not as easy”.

Our participants also discussed the expectations, opinions, and perceptions about the online modality they had prior to (12/13) and following the transition (13/13). Before starting TH, only four therapists were optimistic about the new format, two were neutral, and ten had expressed negative beliefs about TH and its effectiveness. While some participants said that they were “not super concerned about [TH]” (P7) or were even “in favor of it” (P6), Participant 5 admitted:

I was like, how is this gonna work? Is this gonna work? Is this going to harm my clients? Is this going to be helpful to them? … I had no idea, but obviously, my inclination was [that] this might not work very well, this might not be as effective. My clients might really hate this format, I just have no idea.

Another participant (P3) shared their beliefs before switching to it themselves: “I guess I didn’t super worry about effectiveness because I just kind of had the assumption that people who are doing teletherapy weren’t very good therapists”.

Interestingly, our data revealed a new typical pattern among participants after the transition, with most participants expressing mixed reactions. While nearly all participants (12/13) became more positive about TH and found it effective, 11 of the same group also voiced negative aspects, and 10 maintained a neutral stance. For instance, Participant 4 shared, “My beliefs have changed, and this experience has made me believe in the ability to be effective in [TH]. I genuinely believe that my clients are benefiting from teletherapy. It doesn’t feel to me like … [a] second rate type of therapy”. However, they also noted, “I never feel as connected as … in-person”. While they felt this did not greatly impact the effectiveness of their service, they added they would not want to exclusively practice teletherapy.

Another therapist (P5) emphasized the importance of allowing clients to choose the format that worked best for them, highlighting the value of being able to “meet that client where they’re at”. Also, while Participant 8 felt TH was “an inferior service to in-person”, Participant 11 believed that teletherapy can be just as effective as in-person therapy, especially among cognitive therapists, “I think for a cognitive behavioral therapist, there’s … nothing you can do in person that you can’t do online”.

Our data identified another general category discussed by all participants: the changes in energy levels required for conducting TH sessions. Eleven therapists reported that TH required more energy from them, indicating that the demands can be taxing. Participant 11 explained:

I think [TH] is definitely more taxing. … My wheels in my head are just spinning about: what to do next, say next, how to interpret what I’m hearing and seeing on the screen. And I feel like I took a lot more for granted about those interactions and could be more emotionally connected with a client [in-person]. And that emotional connection is also energizing, uplifting for me, whereas as the cognitive labor that I’ve engaged in right now, it’s really hard.

Other participants in this category shared similar sentiments, saying that TH “is not as … exciting or easy to focus” (P2); it required “more effort for the same, or maybe even slightly less [effectiveness] than what you would get in person” (P10); “it’s definitely more draining” (P6); and “it’s more focused [and] you’re just trying to pick up on more and really trying to study and see if there’s anything is being missed” (P9).

Meanwhile, five therapists stated that TH used the same amount of energy as traditional therapy, viewing it as just different rather than more demanding. For instance, Participant 3 summarized their experience:

I think it’s harder for me to focus because there is not a human in front of me, and that is easier for me to focus on than the screen. … I make a habit of being distracted and not paying attention to screens, you know. And so, I think in session, it’s probably more difficult to stay as focused on my client as I need to. But I don’t know that I find it any more tiring than the amount of energy I spend focusing on my client [when in person] because I have more cues, I have more data in the room… I think … I’m trading drains and gains. … Net neutral.

Only one therapist (P7) reported that communicating with clients through the screen made them feel “a little less tired from [TH]”.

## Discussion

This study sought to understand and describe the therapist’s experience transitioning to TH during the COVID-19 pandemic. The results differ from previously published studies in several key aspects but also replicate some of the findings. Interestingly, the therapists in our sample—in a US-based university counseling center and working within a particular community—have similar experiences to therapists embedded in different contexts, suggesting that these experiences may be more generalizable than expected.

Access was a significant element in our findings, emphasizing that when clients are physically ill or located far from the clinic, TH presents a distinct advantage. Similar to the findings of other qualitative analyses, many therapists were surprised to find that the new format appeared to be an effective treatment for their clients (see Aafjes van Doorn et al., 2024). The format of TH as an accessible option has shown to be especially important for young adults, which form the major clientele in university counseling, even as other groups (rural, older adults, low-resourced groups) may continue to find even this option carrying barriers (see Olfson et al., 2024).

Our therapist participants spoke not only about client fit for TH but also their own suitability to the format. For example, we noted that many therapists working from home had to compete with the demands of families, pets, unilaterally managing logistics and technology, and other challenges. We observed that attention difficulties can be exacerbated when working remotely, with the distractions at home. The person of the therapist as they make such a significant adaptation presents a direction for continuing research, as recommended by Fernández-Álvarez and Fernández-Álvarez 2021. The burdens of caring for clients in the midst of intense emotional demands can be seen in this crisis and may shed light for future widespread crises.

Our participants emphasized the importance of assessing whether the therapy clients are a good fit for the TH format. While some therapists see clients with autism spectrum concerns, agoraphobia, or trauma as an appropriate or even an especially good fit, other therapists argue that clients with autism spectrum and trauma are not well suited to TH. A related study (Aafjes van Doorn et al., 2024) found therapists seeing advantages for TH in working with autism spectrum, trauma, agoraphobia, or even eating disorder concerns. We found similar overlap in findings related to working with clients experiencing suicidality, domestic violence, and psychosis or mania, all of which were seen by therapists as not appropriate for TH. The need for formal guidance from the discipline around treatment of more severe clinical presentations within TH has been noted by Fernández-Álvarez and Fernández-Álvarez 2021 and our therapists experienced the uncertainty in this domain.

Legal and ethical concerns featured prominently in our participants’ experiences, with therapists expressing concerns about unethical practices and compromised patient care due to insufficient training and lack of competency to deliver effective mental health services via TH. Training in TH-specific practices and interventions can be remarkably effective, as the field is now demonstrating (see Lin et al., 2025). Given our therapists’ transition without such training, the adaptations were more challenging. Similar to our findings, Aafjes van Doorn et al. (2024) raised legal and ethical concerns, emphasizing the need for clear guidelines on HIPAA compliance, insurance, and practice across state borders. Privacy and confidentiality were common concerns, with both studies stressing the importance of secure digital platforms, data protection regulations, and educating clients about their shared role in maintaining privacy. The ethical concern shared by therapists emphasized how TH could—counterintuitively—create barriers to equitable access for individuals without tech resources or safe environments (see Olfson et al., 2024).

However, our study revealed additional therapist privacy concerns not thoroughly examined in other studies, as they emphasized the experiences as impactful to themselves as therapists. Our participants emphasized the disadvantages of TH opening a window into clients’ personal environments, voicing their discomfort about involuntary disclosure of their own personal spaces, and noting the blurring of professional and personal boundaries. Another ethical concern was setting communication boundaries with clients, such as clients contacting therapists via email or personal cell phone numbers for non-schedule-related issues. Furthermore, participants in our sample faced an ethical challenge-one that seems especially relevant for work in university counseling centers—when it comes to receiving crisis messages during off hours. This created a dilemma between maintaining a work–life balance and addressing clients’ urgent needs.

Our findings also highlight how therapy itself, including therapeutic attitudes, interventions, and interactions, shifted when conducted in a TH format. Therapists in our sample pointed to their experiences in ways consistent with other studies (see Békés et al., 2023), including that they became more directive with their clients, that they lacked access to non-verbal cues, and that these contributed to feeling less connected and finding it more difficult to understand their clients. However, the therapist participants found innovations in their TH work, such as finding ways to use the client’s living environment in real-time. While a minority of therapists indicated there was little change in their therapy processes in TH, 12 of the 13 therapists also reported they were able to engage in the session and effectively utilize interventions in TH with similar effectiveness as in-person services, despite the many negative impacts as were described by the same participants. As in other studies, a majority of therapists noted that TH leads to reduced intimacy (Békés et al., 2021, 2023; Full et al., 2024) and lower energy (Aafjes van Doorn et al., 2022; McCoyd et al., 2022; Sampaio et al., 2021) for the therapist compared to in-person sessions. Our therapists found an overall similarity to the findings summarized by Fernández-Álvarez and Fernández-Álvarez 2021 that while adaptation is needed, the therapeutic relationship can still be established via TH. The increased sense of disconnection due to limited non-verbal cues, with the rare exception of some therapists reporting increased connection with specific clients, remains a matter for creative solutions and improved training in the TH modality (see Lin et al., 2025).

The diminishing of therapist energy when using the TH format deserves attention, as the majority of therapists in our sample reported this impact while noting that the TH format was effective. These findings parallel those of McCoyd et al. (2022), with the additional overlap that therapists in both studies identified the screen as a barrier to connection (i.e., restricted field of view, temporal and physical distance, etc.). With regard to these experiences, the therapy relationship felt better and easier to maintain for these therapists with their clients whom they had first met in person before transitioning to TH, compared to clients whom they had only met through TH. Therapists in both studies also reported a theme of missing the in-person energy of face-to-face sessions and some difficulty creating new professional boundaries that became more fluid during the pandemic context (e.g., how to effectively contact clients outside of session).

The shift in perception for therapists as they adopted TH was also notable. Therapists reported having a negative perception of TH before practicing it, then after practicing it for some time, they found it effective even if personally draining and distracting, paralleling experiences described in other studies (Aafjes van Doorn et al., 2022). Adopting a change requires adaptation and therapists’ shifts in attitudes may parallel similar shifts in clients’ attitudes when adopting the technology (see Fernández-Álvarez & Fernández-Álvarez, 2021).

Overall, our findings highlighted the experience of university counseling center therapists, whose positive and negative experiences with TH emphasized the nature of work in this setting. Access and equity, ethical dimensions of fitness for TH, and the need for boundaries with work and clinical practice appear especially pronounced when the providers are responding to the mental health needs of a specific community. Our findings also highlight that working within a short-term model makes TH advisable in some ways (ease of access) and less appealing in others (difficulty assessing for non-verbal cues, shift away from some interventions). Fitness for TH interventions becomes an even more essential topic for these therapists in this setting.

We found it helpful to get comprehensive responses from a smaller number of participants to gather detailed and insightful data. This contrasted with some other studies that used large samples of therapists with shorter qualitative responses while being more similar to studies with smaller numbers of therapists. The quotes shared in this manuscript and our full dataset (https://osf.io/5wh74/?view_only=9f31998f663a4b349dc0c662681ed50f) offer an in-depth understanding of these experiences. Finding more overlap in therapist responses within our setting strongly suggests that these aspects of TH practice and transition may be generalizable.

### Implications and future research

The findings underscore the need for comprehensive training to address logistics, ethics, and protocols associated with TH. Many therapists felt unprepared for the transition and highlighted the importance of professional development and peer support. Even experienced therapists reported a lack of confidence and competency in their clinical skills when using the TH format. In light of these challenges, training programs should cover a range of topics, including establishing therapeutic alliances across distances, handling ethical concerns around privacy and confidentiality, and ensuring therapist competence in using online platforms. Inchausti et al. (2020) suggested several key points to guide therapists in adapting their practice to the TH format. For instance, therapists should ensure an appropriate video setup by maintaining clear camera positioning showing at least a half-length shot (vs. face-only view) with some background to enhance engagement and convey natural nonverbal cues through posture and hand gestures. To help normalize the experience and foster connection, therapists should use strategic self-disclosure, such as sharing minor personal challenges with telehealth (Inchausti et al., 2020). Furthermore, Lin et al. (2025) provided empirical evidence demonstrating that targeted training in teletherapy could help mitigate some of the challenges identified in this study, particularly around maintaining connection and engagement.

Furthermore, future efforts should focus on establishing explicit roles and responsibilities for both therapists and clients in managing confidentiality in the TH format, educating clients about the importance of securing a private space for therapy sessions, and providing therapists with strategies to address potential confidentiality issues. Inchausti et al. (2020) recommends that clinicians guide clients in creating a secure therapy space, suggest alternative locations like a car or private garden when necessary, discuss potential disruptions (e.g., intrusions by household members or technical failures), and establish backup plans.

These training objectives could be integrated into clinical curricula for training programs and continuous education to better prepare clinicians for TH work. Furthermore, implementing TH effectively involves understanding the specific training requirements that make clinicians comfortable with it and managing therapist fatigue from significant changes. Fernández-Álvarez and Fernández-Álvarez 2021 suggested that training programs should be evidence-based, focusing on general principles of change over manualized treatments to prevent conflicts between therapeutic schools and promote therapeutic competence.

Future research should continue to emphasize the inclusion of larger, more diverse samples to address specific findings from our study. For example, outcome research could explore the long-term effects of TH. Process researchers could examine whether some of the TH adaptations to interventions, identified by our therapist participants, became widespread among practitioners. Specific interventions could be developed within the format, and training programs could examine how to address ethical and logistical concerns best. For instance, Pugh et al. (2021) provided concrete examples of therapists successfully adapting interventions to the TH format by using symbolic objects to modify chair work, adjusting chair placement, and increasing verbal directions. These adaptations demonstrate that experiential techniques can be effectively maintained and even refined in a virtual setting, highlighting the importance of flexibility and creativity in TH rather than abandoning proven methods. Moreover, the ability to repurpose familiar interventions underscores the fact that while telehealth alters the therapeutic process, it also presents opportunities for innovative approaches to client engagement.

Future studies could also investigate how different therapeutic interventions are affected by the format and which therapeutic modalities are most and least impacted. Special attention could be given to understanding the match between certain clients and the TH format to diagnose and manage client crises and suicidality remotely. Fernández-Álvarez and Fernández-Álvarez 2021 discuss the complexities of delivering mental health care services in an online format, emphasizing the importance of tailoring interventions to different modalities, settings, and clients’ individual differences, preferences, and clinical problems. Additionally, a similar qualitative study conducted in a different cultural context (Fernández et al., 2022) found comparable results, suggesting universal core challenges and benefits of TH. Further cross-cultural explorations should focus on how these shared experiences might be shaped by contextual factors and ways to optimize TH across various cultural settings.

While the current study focused on therapists’ experiences, the lack of patient input left a gap in our knowledge of how TH is received, how engagement differs from in-person therapy, and whether patients perceive similar benefits and challenges (Fernández et al., 2022). Future studies should incorporate simultaneous exploration of patients’ perspectives in comparison to those of therapists to address these limitations and gain a more comprehensive understanding of TH. To further expand our knowledge of factors that could influence TH experiences, future research should consider various therapists’ characteristics (e.g., gender, age, personal style, level of experience, and educational degree) and patient characteristics (e.g., age, socioeconomic status, and digital literacy). Understanding these factors could inform the refinement and personalization of TH interventions to better meet patients’ needs and preferences, particularly for populations with barriers to digital access, such as homeless, lower-income, and elderly patients (Markowitz et al., 2021). Olfson et al. (2024) propose several strategies to mitigate these disparities, such as educating patients with limited digital literacy, offering video visits to all patients who can be safely treated in this manner, and addressing financial constraints. Future knowledge on this subject can be solidified by other qualitative replications and qualitative meta-analyses (Levitt, 2018).

### Limitations

This study has several limitations. Consistent with most qualitative research, the sample size is small and not nationally representative in terms of diversity. Additionally, because the sample was drawn from a single university counseling center with primarily white clinicians, the findings may not generalize to more diverse clinical settings. The unique context of the pandemic period adds another limitation to generalizability.

Furthermore, given the small sample size, the diversity in terms of therapists’ age, gender, treatment formats (individual or group), and patient characteristics, including clinical conditions and socio-economic status, introduces variability, making it difficult to isolate common trends in TH experiences. Additionally, we did not collect data on participants’ level of experience or specialized training beyond their education and licensure status. This highlights the need for future studies to examine these factors in larger, more controlled samples. Finally, as is the case with qualitative coding, the use of human coders is limited by the biases present among the coders, although human interpretation remains essential.

## Conclusion

As therapists adjusted to TH, they showed their ability to adapt, work through challenges, and meet the needs of their clients. Their experiences show valuable insights into how to better conduct TH, train for forming therapeutic relationships, and engage in ethical practice. Furthermore, as TH continues to evolve, our study’s findings contribute to the ongoing discourse on its integration into mental health treatment, emphasizing its potential permanence. This knowledge will play a crucial role in shaping the training of a new generation of clinicians, ensuring they are adept at delivering services seamlessly, whether in-person or online, keeping pace with the evolving needs of mental health treatment delivery.

## Data Availability

The complete de-identified analyzed dataset is available at the Open Science Framework (https://osf.io/5wh74/?view_only=9f31998f663a4b349dc0c662681ed50f). This study was not preregistered.

## Appendix A. Interview schedule

1. What is your in-person therapy style? Can you give me a prototypical example of how you do therapy?
2. How has your therapy style been affected by the Teletherapy format? Can you share an example?
3. What is it like to have limited non-verbal cues in a session? How has it affected the therapy?
4. How have your interactions changed in the Teletherapy format? How have they been the same? Examples?
5. How connected do you feel with your clients in the teletherapy format compared to in-person therapy? How is it similar or different from in-person services? Examples?
6. What were some of your beliefs about the effectiveness, difficulty, or experience of teletherapy before you started providing teletherapy services?
7. How have your beliefs about teletherapy services changed or remained the same since starting teletherapy services?
8. How has the location (home vs work office) of providing services affected your experience? Has therapy felt different doing it from an UVU office compared to doing it from a different location? Can you provide examples?
9. In your opinion, which clients are appropriate for teletherapy services? Which clients are not appropriate for teletherapy services?
10. How has the switch to teletherapy services affected your energy levels (Globally and in-session)? Do you feel you have more energy, less energy, or no changes in energy levels? Have you experienced both? Why do you think this is? Examples?
11. How well have your clients adapted to the teletherapy medium? Are they more open or less open? More willing to emote or less willing to emote?
12. Please share a positive experience of teletherapy and what made it such a positive experience for you.
13. Please share a negative experience of teletherapy and what made it a negative experience.
14. How has your availability and communication with clients outside of session changed? How has that been for you? What changes to procedure have been made for this new format?
15. This center switched from in-person therapy to teletherapy rather quickly. What were the most difficult parts of the transition? What were the least difficult parts of the transition?
16. How comfortable were you in transitioning to teletherapy? Have you become more comfortable over time? Why or why not?
17. What ethical concerns have you had when doing teletherapy? How have you handled them?
18. What training would you have like to had before making the transition to teletherapy?
